# Effectiveness of multimedia education on the childbearing intention in one-child women: a field trial study

**DOI:** 10.1186/s12909-024-05784-6

**Published:** 2024-08-25

**Authors:** Nasrin Aghazadeh, Saber Jabbari Farooji, Shima Haghani, Leila Amini

**Affiliations:** 1 Department of Midwifery and Reproductive Health, School of Nursing and Midwifery, Iran University of Medical Sciences, Tehran, Iran; 2 Maragheh University of Medical Sciences, Maragheh, Iran; 3 Ministry of Health and Medical Education, Tehran, Iran; 4 Nursing and Midwifery Care Research Center (NCRC), Iran University of Medical Sciences, Tehran, Iran

**Keywords:** Childbearing intention, Women, Education, Multimedia, One- child

## Abstract

**Background:**

childbearing is a key component of population movements and sustainable development in low-fertility nations. This study was conducted with the aim of determining the impact of multimedia education on the childbearing intention in One-child women of Maragheh city.

**Method:**

In this intervention study in Maragheh in 2023, 94 women with one child were randomly selected and placed in two intervention and control groups. Before the intervention, people were examined using demographic information and Miller’s questionnaire of desire to have children. Then, For the intervention group, three 60-minute multimedia training videos were shown in person for three weeks, and the control group did not receive any training. The data were collected using the researcher’s demographic questionnaire and Miller’s childbearing questionnaire in three stages before the intervention, immediately after and six weeks after the intervention. Data analysis was done with SPSS version 16 software.

**Results:**

There was no significant difference between the two groups in terms of the average score of desire to have children and demographic variables (*p* > 0.05). Before the intervention, there was no significant difference between the intervention and control groups in terms of the demographic characteristics of the subjects and the average score of women’s willingness to have children in the positive and negative dimensions. While after the multimedia educational intervention, the average score of desire to have children in positive and negative dimensions, immediately after the intervention and six weeks after the intervention, had a significant difference compared to before the intervention (*p* < 0.001).

**Conclusion:**

The findings of the study showed that carrying out multimedia educational interventions to single-child women can have a positive effect on their desire to have children. It seems that the implementation of such programs is effective in the conscious decision of families to have children and ultimately increases the intention of the individual to carry out the behavior.

**Trial registration:**

Iranian Registry of Clinical Trials: IRCT20230227057549N1. Date of registration: 16/04/2023. URL: https://irct.behdasht.gov.ir/.

## Background

Childbearing is one of the most basic rights men and women have while living together [1, 2]. Globally, fertility rates have sharply declined, particularly in developing nations, as a result of birth control policy implementation, changes in family attitudes about having children, and changes in demographic patterns during the past several decades [3].

childbearing is one of the most important components of population movements and is the focus of sustainable development in countries with low fertility rates [4] So that childbearing has a high value in all societies [5].

The revision of the family planning legislation in Iran in 2008 marked the initiation of strategies aimed at boosting the population and halting the decrease in fertility rates. The rise in the total fertility rate to 2.01 in the 2015 census initially indicated success in population growth policies. However, the rate has declined since 2016, reaching 1.6 in 2019, suggesting that population policies have not been effective in the past decade [6]. The predictions have also shown that the overall fertility rate among the Iranian population would continue to decrease to a level below 1.5 by 2025 [7].

Population aging is a significant outcome of reduced population growth, resulting in many challenges in production, economic growth, and workforce shortages [8] The employment of foreign workers will lead to the introduction of incompatible subcultures and an increase in moral and social abnormalities [9]. The scarcity of labor will lead to a higher proportion of women participating in the workforce, which will be the primary outcome of the trend of delaying childbirth [10].

An significant topic in the study of fertility is the concept of having a single child [11]. Having only one child and a preference for having only one child can lead to very low fertility rates (less than 1.5 children), which can result in negative population growth, declining population numbers, and a lack of replacement in the future [12].

Being an only child can lead to issues including hindering the child’s growth and development, fostering a self-centered and lonely mentality, and raising the likelihood of anxiety and sadness in these individuals [13]. most of the only children have a lot of dependence on their parents and cannot establish a sincere relationship with their peers. they have elevated reading skills and focus, although also experience increased feelings of dissatisfaction and dependency on their parents [14].

Lack of awareness and a negative viewpoint towards fertility may lead to delayed childbearing and reluctance to expand households, Conversely, a favorable outlook on having children fosters a wish to have offspring [15].

Given this circumstance, it is imperative to implement focused educational initiatives, particularly those facilitated by the media, in order to alter perspectives on childbearing [16]. Over the past few years, multimedia health education has become increasingly prevalent in clinical settings and in the promotion of health care policy [17]. Furthermore, this pedagogical approach supports learners’ indirect learning through the promotion of active engagement in establishing connections between concepts, the facilitation of content repetition for the purpose of reuse, and adaptability [18].

In Iran, numerous studies have examined the various factors that influence childbearing. However, research on the effect of educational interventions in improving fertility knowledge is limited [19, 20].

Vatanparast et al.‘s study at Mashhad University of Medical Sciences aimed to investigate the impact of education on the intention to have children in single-child women using the theory of planned behavior. The study concluded that training based on this theory did not influence the intention of single-child women to have children, suggesting a need for further research in this area [21]. Also, another study was conducted with the aim of determining the impact of the educational program based on the meta-theoretical model on the attitudes and stages of changing the childbearing behavior of women, The results indicated that while the program did improve women’s attitudes toward childbearing, it was unable to alter the stages at which they changed their behavior. And to induce a desire to have children and induce a change in behavior, training that is lengthier in duration, includes a greater number of samples, and employs innovative educational techniques is required [22].

Multiple studies have demonstrated that the dissemination of information via media exerts a substantial influence on the audience’s conduct [17, 23]. In contemporary times, a critical determinant of any society is its citizens’ consumption patterns. This becomes even more imperative in light of the continuous proliferation of virtual media and the Internet, which have accelerated the investigation of the societal impacts of consumption patterns [24]. In addition to school, family, and acquaintances, Gides identifies the media as a significant determinant of socialization, a notion that has gained prominence in contemporary society [23].

Given the significance of multimedia education in elucidating the primary determinants of single-child behaviour and the importance of encouraging childbearing, particularly among single-child women, the current study was conceived. This research was conducted with the aim of the Effectiveness of Multimedia Education on the Childbearing Intention in One-Child Women.

our research hypothesis are as follows:

1. Educational intervention affects the positive and negative aspects of the desire to have children.
2. husband’s employment and duration of marriage would impact the intervention results.
3. The results of the intervention in the stage of 6 weeks after the intervention are reduced compared to the stage immediately after the intervention.

## Methods

### Study design and setting

**Fig. 1:**
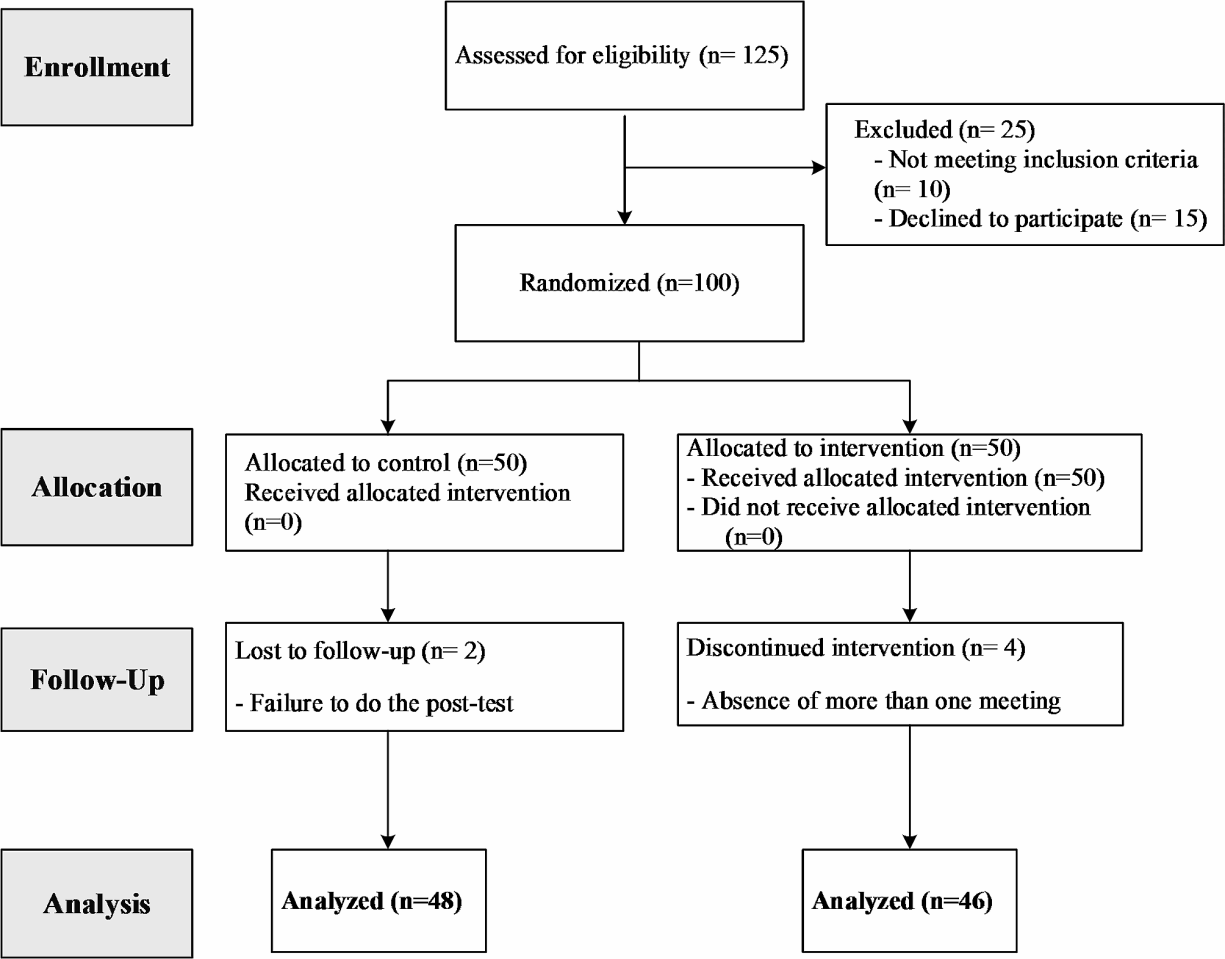
Study CONSORT diagram

The study was a field trial study and adhered to the CONSORT guiding principle (Fig. 1), conducted between April 2023 and June 2023. The research design was in compliance with the guidelines and regulations of the Declaration of Helsinki Ethical Principles for Medical Research involving human subjects and was approved by Vice Chancellor for Research of Iran University of Medical Sciences, Faculty of Nursing and Midwifery with ethics code IR.IUMS.REC.1401.916 and registration code of (IRCT20230227057549N1) on Iranian Registry of Clinical Trials site.

### Sample and sampling technique

This investigation was a field trial study conducted between April 2023 and June 2023 on 94 single-child mothers who met the inclusion criteria and were referred to the health centers of Maragheh City, Iran.

To conduct sampling, the metropolis of Maragheh was partitioned into four distinct regions, namely the North, South, East, and West. South and West regions and North and East regions were similar in terms of socio-cultural and economic level. In addition, in order to reduce the risk of data contamination, using the lottery process, the samples of the intervention group were selected from the north and south regions, and the samples of the control group were selected from the west and east regions. After that, in regions with multiple health centers, a randomization was used to select one center at random from each region (all centers in each region were homogeneous).

Among the eligible women who referred to health centers, sampling was done continuously and in proportion to the number of patients for two months. In this way, 30 people from comprehensive urban and rural health service center number 6, 30 people and from comprehensive urban and rural health service center number 2, 20 people for the intervention group and from the comprehensive urban health service center number 9, 18 people, and from comprehensive urban health service center number 10, 32 people were included in the study for the control group.

The single-child women completed the informed and freely written consent form during sampling in the health centers after being informed of the study’s objectives and method and meeting the inclusion criteria.

Inclusion criteria include Iranian nationality To be married, Having at least a middle school education, having a child aged at least two year (note: Because the maximum care and dependence of the child is up to 2 year old, it is possible that a biased answer may be created in response to the mother’s desire), Age range from 15 to 45 years and the unwillingness to have a second child. Non-entry criteria include Suffering from physical and mental illness that hinders learning, Known fertility problems in husband or wife, contraindications for re-pregnancy, Participation in similar training programs and the occurrence of stressful events such as divorce, death of husband. and exclusion criteria include Absence of more than one session in the educational program.

The sample size based on the study of Vatanparast et al. [21], at the 95% confidence level, and 80% power, According to the statistical drop, 50 subjects for each control and intervention group were determined (*n* = 100). The results of power analysis were equal to 0.629.

### Measuring instrument

The data collecting instrument included a demographic survey and Miller’s childbearing questionnaire.

#### Demographic questionnaire

The 15 queries on the demographic questionnaire assessed individual characteristics and fertility history. Demographic variables (*n* = 11) included age of wife and husband, level of education of wife and husband, ethnicity, employment status of individual and spouse, housing status, gender of first child and duration of marriage. Fertility history items (*n* = 4) included the number of previous pregnancies, history of abortion, history of stillbirth and history of infertility.

#### Miller’s childbearing‑motivation questionnaire

Warren Miller created this 1995 questionnaire with good and negative aspects. There are 28 items on the positive dimension and 21 items on the negative dimension in the original version of this tool. This questionnaire has been psychometrically evaluated and used in different populations. Based on Khadiovazadeh’s research [25], this questionnaire has been modified for Iranian culture in the Persian version. 7 questions were added to Miller’s questionnaire’s positive motives and 2 were combined. In the tool’s negative dimensions, two items were decreased. Miller’s questionnaire has an alpha coefficient of 0.91 for the positive component and 0.94 for the negative dimension of child desire, confirming its internal consistency. The Childbearing Motivation Questionnaire was scored on a 4-point scale from 1 (complete disagree) to 4 (total agree). The current questionnaire that is used in this research has 34 questions on the positive aspects of the desire to have children, including joy of pregnancy, birth, and infancy (6 items); traditional perspective (6 items); childrearing satisfactions (6 items); feeling needed and connected (5 items); as well as instrumental values of children (11 items)0.19 questions on the negative aspects of the desire to have children, including fear of parenthood (7 items), parental stress (8 items), and child care challenges (4 items). Positive scores on the questionnaire range from 34 to 136, while negative scores range from 19 to 76. Higher positive scores and lower negative scores suggest a stronger desire to have children [26].

#### Validity and reliability

Khadivzadeh et al. assessed and validated the validity of the Persian version of this questionnaire. Ten couples completed a reliability assessment of the tool using the test-retest procedure. The correlation between the data in the two stages of the test is equal to 0.98. The internal homogeneity of the data was assessed using Cronbach’s alpha coefficient. The questionnaires’ reliability was validated using alpha coefficients of 0.91 and 0.94, respectively, for positive and negative fertility motivation [25].

In the present study, content validity method were used to validate the data collection tool. In this way, the data collection tool was selected first by studying valid and new articles and with the guidance of professors, then it was given to 3 members of the Faculty of Nursing and Midwifery of Iran University of Medical Sciences in order to collect correction opinions, and the final corrections were made. To determine the reliability of the data collection tool, Cronbach’s alpha method was used on 20 people who were not present in the main study. The questionnaire’s reliability was validated through the calculation of alpha coefficients of 0.89 and 0.91, respectively, for positive and negative fertility motivation.

### Educational intervention

After explaining the study’s goals to the community, describing education and the study’s methods, and ensuring confidentiality, single-child women who met the study’s criteria and signed a written informed consent form were sampled. To avoid inaccurate and impatient study participants due to center crowding, the questionnaire was developed online and given to them. When the research unit required clarification on how to complete the questionnaire, the researcher assisted it via telephone conversation. The meeting times and locations were communicated via text message, and in order to verify participant attendance, a telephone conversation was conducted with each individual one day prior to the training session. In the classroom of the comprehensive center of urban and rural health services No. 6 in the city of Maragheh, which was furnished with computers, projectors, and speakers, educational sessions were conducted. All participants in both groups completed the research questionnaire prior to the intervention.

Participants of both groups completed the Miller Childbearing Intention Questionnaire online in three stages: before the intervention, immediately after the intervention, and six weeks after the intervention. Based on Fig. 1, two people from the control group were taken out of the study in the follow-up phase because they didn’t show up to fill out the third-stage questionnaire, and four people from the intervention group were taken out of the study because they didn’t fully attend the training sessions.

#### Multimedia educational content

In the creation of educational multimedia, the expertise of professors of the midwifery department of Iran University of Medical Sciences and valid population and fertility data, other academic perspectives were used. Educational multimedia was produced based on Mayer’s seven principles.

The data collection process was as follows:

In the first stage (before the educational intervention), without any intervention, both the intervention and control groups completed the questionnaire of demographic characteristics and Miller’s childbearing questionnaire.

In the second stage (intervention), multimedia training was given to the intervention group. In this way, educational multimedia was shown in person once a week for three weeks to the participants of the intervention group. But the control group did not receive any training. After this, both the intervention and control groups completed the Miller’s childbearing questionnaire.

In the third stage (6 weeks after the educational intervention), without any other educational intervention, the Miller’s childbearing questionnaire was given to all the participants of the intervention and control groups and the questionnaire was completed (Table 1).

**Table 1:** Educational intervention program

Meeting title and training technique	session number/time
- Introducing and stating the goals of training sessions - An introduction to the changes in population growth in Iran and new population policies (play multimedia content for 20 min) - Questions and answers (40 min)	First session (60 min)
- The importance and benefits of having children - The effects of having a single kid from the viewpoint of the family and the community - Answers to common questions about parenting (play multimedia content for 20 min) - Questions and answers (40 min)	second session (60 min)
- Injuries of being an only child with the approach of psychology and educational sciences - Expressing problems and obstacles regarding having children - Healthy lifestyle before pregnancy (play multimedia content for 20 min) - Questions and answers (40 min)	third session (60 min)

### Data analysis

The data were subsequently evaluated utilizing SPSS 16 software. The normality of the quantitative data was confirmed using the Shapiro-Wilk and Skewness tests. Descriptive and inferential statistical tests, such as the chi-square test, Fisher exact test, independent t test, repeated measures analysis, and independent samples test, were applied to the data.

## Results

94 women with one child were studied in the intervention and control groups. In both the control and intervention groups, housewives comprised the majority of the participants. And in terms of education level, the majority of the women in the intervention group and the control group had a diploma or higher, and the education level of their husbands was also university. There wasn’t a significant difference in the studied variables between the intervention and control groups, and the two groups were homogeneous regarding demographic data and past obstetric history (*p* < 0.05). demographic characteristics can be seen in Table 2.

Variance analysis of repeated data for variables with normal distribution was used to investigate the effect of training and compare the change in the score of theoretical constructs in two intervention and control groups in three periods (before the intervention, immediately after the intervention and six weeks after the intervention).

Based on the results of the independent t-test, The positive motivation score was not significantly different between the intervention and control groups before, immediately, and six weeks after the intervention (*p* > 0.05). Examining the trend of changes in the average scores of the positive motivation of the desire to have children in single-child women in the intervention and control groups at different times showed the average score of desire to have children in the post-intervention phase and six weeks after the intervention was different in the intervention group (*p* = 0.012). However, in the control group, changes in the positive motivation of desire to have children were not significant (*p* = 0.748) (Table 3).

The negative motivation score was not significantly different between the intervention and control groups before, immediately, and six weeks after the intervention (*p* > 0.05). Examining the trend of changes in the average scores of the negative motivation of the desire to have children in single-child women in the intervention and control groups at different times showed that the average score of desire to have children in the post-intervention phase and six weeks after the intervention was different in the intervention group (*p* = 0.009). However, in the control group, changes in the negative motivation of desire to have children were not significant (*p* = 0.300) (Table 3).

In the following, by using Bonferroni’s post hoc test, the process of two-by-two changes in the mean scores of the positive and negative dimensions of desire to have children in single-child women in the intervention group was investigated at different times.

The results regarding the positive motivation show that the trend of the mean scores of desire to have children increased significantly immediately after the intervention (*p* < 0.001). But 6 weeks later, compared to the stage immediately after the intervention, it has decreased significantly (*p* = 0.032), but it is still significantly higher than the stage before the intervention (*p* = 0.022) (Table 4).

The results regarding the negative motivation show that the trend of the mean scores of desire to have children increased significantly immediately after the intervention (*p* < 0.001). But 6 weeks later, compared to the stage immediately after the intervention, it has decreased significantly (*p* = 0.013), but it is still significantly higher than the stage before the intervention (*p* = 0.014) (Table 4).

**Table 2:** Demographic characteristics in both intervention and control groups before the intervention (*n* = 94)

Variable		standard deviation ± X / number (percentage)		*P*
		Intervention	control	
Education	intermediate	9 (19.6)	12 [25]	X^2^ = 1.088 df = 2 *P* = 0.580^*^
	Diploma	18 (39.1)	21 (43.7)	
	College	19 (41.3)	15 (31.3)	
husband’s education	illiterate	1 (2.2)	2 (4.2)	*P* = 0.541^**^
	intermediate	5 (10.9)	6 (12.5)	
	Diploma	12 (26.1)	18 (37.5)	
	College	28 (60.9)	22 (45.8)	
Employment	employed	13 (28.3)	16 (33.4)	X^2^ = 0.283 df = 1 *P* = 0.595^*^
	housewife	33 (71.7)	32 (66.6)	
husband’s Employment	freelance job	22 (47.8)	35 (72.9)	X^2^ = 1.810 df = 2 *P* = 0.048^*^
	Employee	19 (41.3)	8 (16.7)	
	manual worker	5 (10.9)	5 (10.4)	
residence	private house	28 (60.9)	34 (70.8)	X^2^ = 1.039 df = 1 *P* = 0.308^*^
	Rental	18 (39.1)	14 (29.2)	
nationality	Turkish	39 (84.8)	46 (95.8)	*P* = 0.087^**^
	Persian	7 (15.2)	2 (4.2)	
The gender of the first child	Boy	30 (65.2)	28 (58.3)	X^2^ = 0.471 df = 1 *P* = 0.492^*^
	Girl	16 (34.8)	20 (41.7)	
History of stillbirth	Yes	2 (4.3)	1 (2.1)	*P* = 0.613^**^
	No	44 (95.6)	47 (97.9)	
History of abortion	Yes	3 (6.5)	5 (10.4)	*P* = 0.715^**^
	No	43 (93.5)	43 (89.6)	
Age		31.63 ± 6.51	32.12 ± 7.06	t=-0.352 df = 92 *P* = 0.725^***^
husband’s age		36.86 ± 5.85	35.77 ± 6.04	t = 0.895 df = 92 *P* = 0.373^***^
duration of marriage		5.10 ± 1.21	5.68 ± 1.66	t=-1.918 df = 92 *P* = 0.06^***^

^* Chi−square test^

^** Fisher exact test^

^*** Independent t test^

**Table 3:** Mean ± standard deviation of theoretical structures, before, immediately and six weeks after the educational intervention in the intervention and control groups

Structure		Before intervention	Immediately after the intervention	6 weeks after the intervention	Intra-group test result Sig.
Positive motivation	Intervention	96.13 ± 18.89	99.41 ± 18.95	97.80 ± 19.24	F = 6.807^*^ *p* = 0.012 $\:{\eta\:}^{2}$=0.131
	control	97.66 ± 21.16	97.33 ± 20.42	97.81 ± 19.21	F = 0.105^*^ *p* = 0.748 $\:{\eta\:}^{2}$=0.002
	Inter-group Test Sig.	t =-0.371^**^ *p* = 0.712	t = 0.510^**^ *p* = 0.610	t =-0.002^**^ *p* = 0.998	
Negative motivation	intervention	47.26 ± 7.38	44.54 ± 7.47	45.95 ± 7.39	F = 7.529^*^ *p* = 0.009 $\:{\eta\:}^{2}$=0.143
	control	47.85 ± 8.27	47.75 ± 8.17	47.54 ± 7.83	F = 1.100^*^ *p* = 0.300 $\:{\eta\:}^{2}$=0.023
	Test result	t =-0.366^**^ *p* = 0.715	t =-1.982^**^ *p* = 0.050	t =-1.008^**^ *p* = 0.316	

* Repeated Measures Analysis

** Independent Samples Test

**Table 4:** Pair by pair comparison of the changes in the mean scores of the positive and negative motivation of desire to have children in women with one child in the intervention group

Structure		Time	difference in averages	95% CI		Coefficient Standard Error	p-value*
				Lower	Upper		
Positive motivation	Before intervention	Immediately after the intervention	-1.475 -1.475	-2.07	-0.87	0.301	< 0.001
		6 weeks after the intervention	-0.910	-1.68	-0.13	0.389	0.022
	Immediately after the intervention	6 weeks after the intervention	0.565	0.51	1.07	0.259	0.032
Negative motivation	Before intervention	Immediately after the intervention	1.411	0.83	1.99	0.238	< 0.001
		6 weeks after the intervention	0.808	0.13	1.48	0.278	0.014
	Immediately after the intervention	6 weeks after the intervention	-0.602	-1.10	-0.10	0.206	0.013

## Discussion

The present study found that single-child women’s desire to have children increased significantly following the multimedia educational intervention. The educational intervention affected the positive and negative aspects of the desire to have children, so that the score of the desire to have children in the intervention group increased immediately after the intervention and six weeks after the intervention.

Many studies have been conducted regarding childbearing and the effect of education on the desire to have children has been reported in the studies [25, 27]. The results of the study by Akbarian-Moghadam et al., which was conducted with the aim of determining the impact of training based on the theory of planned behavior, It showed that the changes in the desire to have children from the stage before the intervention in the intervention group, immediately after the training and one month after the training, were significantly more than the control group [28], which is consistent with the results of the present study. Also, Williamson et al., in a study that aimed to determine the effect of providing fertility information on fertility knowledge and the intention to delay childbearing, They showed that giving information to people in the field of fertility had an effect on their desire [29]. Alami et al.‘s research examined the influence of the theory of planned behavior-based educational intervention on single-child women’s reproductive intention. The training programme significantly increased the desire score of experimental group women to have children [27]. In another study conducted by Ansari Majd et al. to investigate the effect of a training program based on a meta-theoretical model on the attitude and stages of childbearing behavior change, the results showed that training leads to improvement of women’s attitude and willingness to have children [22]. With regard to the impact of education on childbearing, the findings of the aforementioned studies are consistent with those of the current investigation. Considering the effectiveness of multimedia educational intervention, it seems that it is possible to change people’s attitudes and mental norms towards having children by identifying negative thoughts and distinguishing them from reality and showing happy parents with babies through videos and slides [30]. In order to achieve this objective, the implementation of innovative and sophisticated pedagogical approaches, such as multimedia instruction, can significantly enhance individuals’ knowledge, attitudes, and achievements across various disciplines [31, 32].

Studies have contrasted speech education and multimedia education with one another. Research in different fields has shown the effectiveness of multimedia approaches over more conventional approaches like lectures. According to a research by Norouzi et al. comparing the effects of teaching on two speech and multimedia approaches in the context of employing a communication model, the multimedia method has a stronger impact on performance, attitude, and quantity of knowledge than the lecture method [33]. In a another research, students’ ability to communicate effectively was more positively impacted by the use of multimedia teaching techniques than by more conventional techniques like lectures [34]. Multimedia education seems to be more effective than other teaching strategies in enhancing attitudes, encouraging good mental norms, and raising women’s empowerment levels, all of which increase women’s reproductive intentions and desire to procreate.

Creating a desire and a positive attitude towards having children will lead to having children earlier and with a larger number. While the desire and negative attitude in this regard is one of the factors that reduce the attempt to get pregnant. At the same time, the first important factor in the formation of intention and decision for fertility is attitude, and positive attitude is a prerequisite for positive intention for having children [35]. Hence, educational interventions and measures aimed at encouraging women’s intention to become pregnant should integrate an emphasis on this matter into their framework.

according to Vatanparast et al. The educational intervention did not significantly change the scores of attitude, mental norms, and perceived behavioral control on the intention to have children in single-child women [21], which is not consistent with the results of the present study. It appears that social, cultural, religious, and particularly economic conditions of the society are also influential in altering attitudes toward reproduction, in addition to educational interventions, which should be taken into account for improved outcomes.

Based on the results of the present study, the average score of the positive dimension of the desire to have children in the intervention group after increasing in the stage immediately after the intervention; In the stage of six weeks after the intervention, there was a relative decrease But the average score of six weeks later was still higher than before the intervention. that these changes were also true in the negative dimension. It seems that between the stage immediately after the intervention and six weeks after the intervention, the rapid effect of the education became weaker and other factors also had an impactTherefore, it can be said that having children is a multifactorial category that is associated with many challenges, And a combination of cultural, economic and social characteristics play a decisive and meaningful role in predicting the probability of having children among women [36].

One of the most significant decisions a couple can make is whether or not to have children? Different studies have investigated different reasons and perspectives for the desire to have children. For example, in a study by Lee and Fan, the factors influencing the decision to have children were the need for a fixed income and the costs of raising children, the child’s sexual preferences, and the parents’ health [37].

Employment and occupational group desire to have children and creating a distance between them, strengthening job and financial security, social support for women to have children using the insurance system and favorable working conditions during pregnancy and after that affect the desire to have children [38]. As a result, in order to avert a further decline in fertility, demographic policies should prioritize quality of life and family employment, with a particular emphasis on infertile women, single-child women, and newlywed couples [21].

Additionally, one of the factors that influence childbearing is the age of marriage. The fertility rate is correlated with the age of the couple and the age at which they enter into matrimony, according to the findings of the studies. Pregnancy is currently deferred until the conclusion of a woman’s reproductive period, for a variety of reasons, including an increase in the age of marriage and a rise in the social activities of women. Unfortunately, without taking any measures to get pregnant and without informing the couple about their fertility, the golden years of a woman’s fertility will pass [39, 40].

Abbasi Shawazi and Khawaja Salehi found that women’s desire to have children decreases with education and social activity. Because entering university and increasing education through changes in individual attitudes and beliefs and modern attitudes with increasing age of marriage and delay in childbirth affects fertility [36]. On the other hand, there is a positive and significant relationship between legal protection and working women’s desire to be fertile. Therefore, if population policies can implement programs that match the mother’s role with women’s continued education after marriage, it can be much more effective [41].

Research has indicated that the likelihood of a woman becoming pregnant in the near future is influenced by the perspective of her acquaintances and family members [42]. As the positive effects of education will inevitably have an impact on other members of the family or society at large, it is critical that educational interventions give close attention to this aspect [43].

Based on the information provided, the factors that determine fertility desires in single-child women can be greatly influenced by different demographic and personal characteristics, including religion, nationality, general life values, attitude towards not having children, age, education, housing, and income.

## Conclusion

Multimedia educational interventions and the provision of necessary information to single-child mothers may effectively influence their desire to have children, according to the findings of the present study and the population policies of the country.

It appears that the implementation of such programs influences families’ decisions to have children in an informed manner. The intention of the individual to perform the behavior can be increased, on the other hand, through the use of modern and innovative techniques, such as multimedia techniques and the capacity of virtual space. Thus, researchers propose the use of multimedia education in educational programs pertaining to population growth policy and the development of interventions aimed at promoting couples to procreate.

The efficacy of the present intervention implies that there is room for further expansion of premarital programs and interventions. This demonstrates that the existing procedure fails to align with demographic policies and fails to address the genuine requirements of premarital couples. Given the significance of having children within the family system and society, interventions should aim to enhance attitudes and behavioral intentions in this domain.

Considering the alarming situation of population growth in Iran and the prevalence of single children, it seems that a multimedia educational program for childbearing, such as the one investigated in this study, can effectively increase the desire to having children among single-child Iranian women.

### Limitations

In this investigation, we implemented measures to mitigate possible sources of bias and assure the integrity of our results. In order to reduce any potential distortion in our measurements, we used well recognized and verified tools for gathering data. In order to reduce the likelihood of information bias, the researcher responsible for overseeing the training sessions received comprehensive training to guarantee uniformity and precision in carrying out the study processes. Participants were given explicit instructions to ensure their active involvement throughout the whole study procedure and to offer truthful and impartial responses.

Since the researchers intended to select a real sample from the community, the participants were sampled from different areas of the city, however, selection bias may have occurred. We solved this problem to some extent by using the lottery and randomization process.

Not intervening in the education of crucial individuals, including spouses, parents, and acquaintances, constituted one of the study’s limitations. It is recommended that future research endeavors incorporate essential interventions targeting the demographic groups that impact women’s inclination to conceive, in order to address this limitation.

## Data Availability

The datasets used and/or analyzed during the current study are available from the corresponding author on reasonable request.
